# Polythiophenes with Cationic Phosphonium Groups as Vectors for Imaging, siRNA Delivery, and Photodynamic Therapy

**DOI:** 10.3390/nano10081432

**Published:** 2020-07-22

**Authors:** Laure Lichon, Clément Kotras, Bauyrzhan Myrzakhmetov, Philippe Arnoux, Morgane Daurat, Christophe Nguyen, Denis Durand, Karim Bouchmella, Lamiaa Mohamed Ahmed Ali, Jean-Olivier Durand, Sébastien Richeter, Céline Frochot, Magali Gary-Bobo, Mathieu Surin, Sébastien Clément

**Affiliations:** 1 IBMM, University of Montpellier, CNRS, ENSCM, 34093 Montpellier, France; laure.lichon@umontpellier.fr (L.L.); christophe.nguyen@umontpellier.fr (C.N.); denis.durand@umontpellier.fr (D.D.); lamiaa.ali@umontpellier.fr (L.M.A.A.); 2 Center of Innovation and Research in Materials and Polymers (CIRMAP), University of Mons—UMONS, 20 Place du Parc, 7000 Mons, Belgium; clement.kotras@umons.ac.be (C.K.); mathieu.surin@umons.ac.be (M.S.); 3 ICGM, University of Montpellier, CNRS, ENSCM, CC1701, Place Eugène Bataillon, 34095 Montpellier, France; karim.bouchmella@umontpellier.fr (K.B.); jean-olivier.durand@umontpellier.fr (J.-O.D.); sebastien.richeter@umontpellier.fr (S.R.); 4 Laboratoire Réactions et Génie des Procédés (LRGP), UMR 7274, Université de Lorraine, CNRS, 54000 Nancy, France; Bauyrzhan.myrzakhmetov@univ-lorraine.fr (B.M.); Philippe.Arnoux@univ-lorraine.fr (P.A.); celine.frochot@univ-lorraine.fr (C.F.); 5 NanoMedSyn, 15 Avenue Charles Flahault, 34093 Montpellier, France; morgane.daurat@umontpellier.fr; 6 Department of Biochemistry, Medical Research Institute, University of Alexandria, Alexandria 21561, Egypt

**Keywords:** combined therapy, conjugated polyelectrolyte, imaging, photodynamic therapy, polythiophenes, siRNA delivery

## Abstract

In this work, we exploit the versatile function of cationic phosphonium-conjugated polythiophenes to develop multifunctional platforms for imaging and combined therapy (siRNA delivery and photodynamic therapy). The photophysical properties (absorption, emission and light-induced generation of singlet oxygen) of these cationic polythiophenes were found to be sensitive to molecular weight. Upon light irradiation, low molecular weight cationic polythiophenes were able to light-sensitize surrounding oxygen into reactive oxygen species (ROS) while the highest were not due to its aggregation in aqueous media. These polymers are also fluorescent, allowing one to visualize their intracellular location through confocal microscopy. The most promising polymers were then used as vectors for siRNA delivery. Due to their cationic and amphipathic features, these polymers were found to effectively self-assemble with siRNA targeting the luciferase gene and deliver it in MDA-MB-231 cancer cells expressing luciferase, leading to 30–50% of the gene-silencing effect. In parallel, the photodynamic therapy (PDT) activity of these cationic polymers was restored after siRNA delivery, demonstrating their potential for combined PDT and gene therapy.

## 1. Introduction

Cancer is a multifaceted and complex disease, which remains one of the main causes of death, forcing researchers to continuously develop new anticancer treatments with high efficiency and specificity [1,2]. Most current types of cancer treatments based on chemotherapy and radiotherapy, although beneficial, present multiple side effects including toxicity, non-targeted damage of tissues, neurotoxicity or multidrug resistance [3,4]. Today, photodynamic therapy (PDT) is considered as an interesting alternative to these treatments. Indeed, this therapeutic approach is highly localized and minimally invasive [5]. PDT uses photosensitizers (PSs), which generate reactive oxygen species (ROS) from molecular oxygen under light illumination and thus can directly lead to the cancer cell death mainly through apoptosis or necrosis, and indirectly by inducing tumor vasculature shutdown and recruitment of immune mediators [6]. However, this operational dependence in oxygen in a tumor microenvironment can lead to tumor hypoxia, trigger the signaling cascade mediated by hypoxia inducible factor (HIF) and release pro-angiogenic growth factors, which are ultimately responsible for cancer cell survival or tumor regrowth [7]. To enhance its therapeutic efficiency, the development of oxygen-independent therapeutic approaches is required to collaborate with PDT [8,9,10,11,12,13].

In this respect, gene silencing using small interfering RNA (siRNA) to block gene expression in a highly sequence-specific manner represents a valuable therapeutic approach to combine with PDT [14,15]. Notably, the combination of PDT with silencing critical cancer-associated proteins using siRNA has led to increased PDT efficacy and a remarkable antiproliferative effect [16,17,18,19,20]. To combine PDT with gene delivery, cationic amphiphilic systems based on a hydrophobic PS embedded into inorganic nanoparticles [14,21,22,23] or cationic micelles [13,15,24] are mostly studied. However, these nanosystems can suffer from several drawbacks, such as an undesirable separation between a PS and a carrier avoiding a synergistic effect or the possible aggregation of the PS leading to a reduced generation of ROS [25,26]. For these reasons, alternative multifunctional systems that induce siRNA release and cooperate with robust PDT are still required.

In this context, conjugated polyelectrolytes (CPEs), i.e., polymers with a π-conjugated backbone and bearing ionic flexible side chains [27], have emerged as promising systems for such purpose. Indeed, the presence of the ionic side groups enables their dissolution in aqueous media and their interaction with biologically relevant targets such as DNA [28,29,30,31,32,33]. Besides, their conjugated backbone determines their intrinsic optical properties, such as absorption, fluorescence, and light-harvesting ability, enabling their use in the development of chemo- and biosensors, as well as in bioimaging applications [34,35,36,37]. Exploiting all these versatile features, CPEs were exploited as imaging systems and gene delivery vehicles exhibiting low toxicity and good photostability, together with high delivery and transfection efficiencies [38,39,40,41,42]. In particular, their ability to generate ROS under light illumination has been proved to be a tool of choice in the release of nucleic acids from the endolysosomal compartment to the cytoplasm by destabilizing their membranes and fostering the lysosomal escape [40,42,43,44]. Recently, Fan et al. even reported the combination of PDT with siRNA release in photo-induced charge-variable CPE brushes that encapsulate up-conversion nanoparticles leading to a cumulative antitumor effect between these two therapeutic approaches [45].

Herein, the therapeutic potential of cationic polythiophene-based CPEs as a multifunctional platform in order to combine PDT with siRNA delivery is described. Cationic polythiophenes possess suitable features for this purpose, such as an important affinity for nucleic acids as well as the ability to photosensitize oxygen [43,44,46,47,48,49]. However, water-soluble polythiophenes are generally prepared through a step-growth polymerization method, the resulting structural defects of which affect both the optical properties (absorption, fluorescence) and DNA binding/condensing ability [43,47,48,49]. The availability of a versatile synthetic strategy, e.g., the Kumada Catalyst-Transfer Condensative Polymerization (KCTCP) enables to overcome such drawbacks through a high degree of control over the final structure and molecular weight [50,51]. As such, a detailed investigation of structure–property relationships, notably the effect of molecular weight, an important parameter when considering gene delivery applications influencing both cytotoxicity, complexation and transfection efficiency, can be performed [52,53,54]. In this work, a series of phosphonium-based conjugated polythiophenes with different molecular weights (11.5 kDa to 53 kDa) was synthesized. The choice of phosphonium head group was motivated by the promising potential of phosphonium-containing polymers compared to their corresponding ammonium analogues in gene delivery applications [55]. Indeed, the more localized positive charge distribution at the P atom is supposed to be the origin of the higher nucleic acid binding ability [56]. Studies also revealed that phosphonium-containing polymer vectors result in lower cell toxicity and increased transfection efficiency with respect to their ammonium analogues [56,57]. The photophysical properties (absorption, fluorescence emission, light-induced generation of singlet oxygen) of the synthesized regioregular cationic polythiophenes were found to be dependent on the molecular weight. Their fluorescent properties were exploited for imaging to determine their intracellular location. Finally, their potential to deliver genetic material and act as a PS in a cooperative manner was examined. 

## 2. Materials and Methods

### 2.1. Polymer Synthesis and Characterisation

Poly(3-(6′-bromohexylthiophene)) (**P3HT-Br**) precursors were synthesized according to reported procedures [31,58]. The polymer characteristics (number averaged (M_n_) and weight-averaged (M_w_) molecular weights and the molecular weight distribution (Ð)) of **P3HT-Br1** were estimated by size exclusion chromatography (SEC). An Agilent liquid chromatograph integrating an Agilent degasser (Diegem, Belgium), an isocratic HPLC pump with chloroform (CHCl_3_) as an eluent at a flow rate of 1 mL min^−1^, an Agilent autosampler (loop volume = 100 μL) with a polymer concentration in a solution of 2 mg mL^−1^, an Agilent-DRI refractive index detector (Diegem, Belgium), and three columns (a PL gel 5 μm guard column and two PL gel Mixed-B 5 μm columns (linear columns for separation of M_w_ of polystyrene (PS) ranging from 200 to 4 × 10^5^ g mol^−1^)) was used. For the two other poly(3-6′-bromohexyl)thiophene) precursors, SEC analyses were performed in THF at 35 °C. A Polymer Laboratories liquid chromatograph was used. This chromatograph integrates a PL-DG802 degasser, an isocratic HPLC pump LC 1120 (flow rate = 1 mL min^−1^), a Marathon autosampler (loop volume = 200 mL) with a polymer concentration in a solution of 1 mg mL^−1^, a PL-DRI refractive index detector, and three columns: a PL gel 10 mm guard column and two PL gel Mixed-B 10 mm columns (linear columns for the separation of M_w_ PS standards ranging from 500 to 10^6^ Da). Calibration was perfomed in both cases with polystyrene standards. 

### 2.2. UV-Visible Absorption and Emission Properties

Absorption spectra were measured on a UV-3600 UV-visible double beam spectrophotometer using Software UVProbe 2.33 (SHIMADZU, MARNE LA VALLEE, France). Fluorescence spectra were measured on a Fluorolog FL3-222 spectrofluorimeter using Software DataMax 2.20 (HORIBA Jobin Yvon, Longjumeau, France). The spectrofluorimeter is fitted with a 450 W Xenon lamp, a thermostated cell compartment (25 °C), a UV-visible photomultiplier R928 (HAMAMATSU Photonics, Hamamatsu, Japan) and an InGaAs infrared detector (DSS-16A020L Electro-Optical System Inc., Phoenixville, PA, USA). Excitation and emission beams are diffracted by double-ruled grating SPEX monochromators with the following features: 1200 grooves/mm blazed at 330 nm for excitation and 1200 grooves/mm blazed at 500 nm for emission, respectively. The detection of singlet oxygen emission was performed by using a double-ruled grating SPEX monochromator (600 grooves/mm blazed at 1 µm) and a long-wave pass (780 nm). All spectra were recorded by using 4 faces quartz cells. All the emission spectra (fluorescence and singlet oxygen luminescence) were recorded at the same absorbance (less than 0.2 at the excitation wavelength) by exploiting the lamp and photomultiplier corrections.

Fluorescence lifetime measurements were performed using a pulsed laser diode emitting at 407 nm (LDH-P-C-400M, FWHM < 70 ps, 1 MHz) coupled with a driver PDL 800-D (PicoQuant GmbH, BERLIN, Germany) for excitation and an avalanche photodiode SPCM-AQR-15 (EG & G, VAUDREUIL, Canada) coupled with a 550 nm long-wave pass filter as a detection system for excitation. A PicoHarp 300 module with a 4-channel router PHR-800 (both PicoQuant GmbH, Berlin, Germany) was used for acquisition. The single photon counting method was employed to record the fluorescence decays. The collection of data was performed up to 1000 counts. The data were accumulated in the maximum channel and analyzed using Time Correlated Single Photon Counting (TCSPC) software Fluofit 4.2 (PicoQuant GmbH, Berlin, Germany) based on iterative reconvolution using a Levensberg–Marquandt algorithm, which enables one to obtain multi-exponential profiles (mainly one or two exponentials in our cases).

### 2.3. Singlet Oxygen Measurements

Singlet oxygen lifetimes have been measured exploiting a TEMPRO-01 spectrophotometer (HORIBA Jobin Yvon, Longjumeau, France) equipped with a pulsed diode excitation source SpectraLED-415 (λ_em_ = 415 nm), a cell compartment, a Seya–Namioka-type emission monochromator (600–2000 nm) and a detection system (H10330-45 near-infrared photomultiplier tube with thermoelectric cooler (HAMAMATSU Photonics, Hamamatsuç, Japan). A single photon counting controller FluoroHub-B and the softwares DataStation 2.5.11 and DAS6 6.6 (HORIBA Jobin Yvon, Longjumeau, France) were used to monitor the system.

### 2.4. Dynamic Light Scattering (DLS) and Zeta Potential

Hydrodynamic diameter and zeta potential were estimated using a Zetasizer Nano ZS (Malvern Instruments Ltd., Malvern, UK). The cationic polythiophenes (5 μM) and cationic polythiophenes/siRNA polyplexes at a P^+^/P^-^ ratio of 100 (5 μM) were prepared in water. The measurements were then performed after 30 min incubation at 37 °C to guarantee the complexation of siRNA. Particle size measurements were carried out with transparent ZEN0040 disposable micro-cuvette (40 μL) at 25 °C. Zeta potential measurements were performed at 25 °C and were carried out in DTS 1070 zeta potential cells made with 18 runs for each.

### 2.5. Cell Culture Conditions

Human breast adenocarcinoma **MDA-MB-231,** the standard cell line or this expressing luciferase and red fluorescent protein (MDA-MB-231 Luc RFP) were purchased from ATCC and AMSBIO (Abingdon, UK), respectively. Cells were cultured in Dulbecco’s Modified Eagle’s Medium (DMEM), incorporating 10% fetal bovine serum and antibiotic (0.05 mg mL^−1^ gentamycin). The cell growth was performed in humidified atmosphere at 37 °C under 5% CO_2_.

### 2.6. In Vitro Cytotoxicity Assay

The in vitro cytotoxicity analyses were performed by using MDA-MB-231 cells seeded into 96-well culture plates, 2000 cells per well in 200 µL of culture medium, and allowing them to grow for 24 h. The cells were then treated with different concentrations of phosphonium-based polythiophene conjugated polyelectrolytes, and, after 3 days, a colorimetric assay of living cells (MTT, (4,5-dimethylthiazol-2-yl)-2,5-diphenyltetrazolium bromide, assay) was performed, as previously described [59].

### 2.7. In Vitro Phototoxicity Assay

In vitro phototoxicity assays were performed with MDA-MB-231 cells seeded into 96-well plates at a concentration of ~2 × 10^3^ cells/well in 100 μL of culture medium and allowed to grow for 24 h. The cells were then incubated for 24 h in the presence or absence of polymer (5 μM in water). After incubation with polymers, cells were submitted, or not, to light excitation (λ = 450 nm, 1.45 J cm^−2^) for 5 min. A total of 48 h after irradiation, the cytotoxicity or phototoxicity of the polymers were evaluated using an MTT assay. Briefly, the incubation of cells was performed in the presence of MTT (0.5 mg mL^−1^) during 4 h to determine mitochondrial enzyme activity. Then, the cell medium was aspirated and the MTT precipitates were dissolved in an ethanol/DMSO (1:1) solution (150 μL) and a reading of the absorbance at 540 nm was performed.

### 2.8. Confocal Fluorescent Imaging on Living Cells 

Confocal fluorescent imaging was perfomed on MDA-MB-231 cells seeded onto glass bottom dishes (World Precision Instrument, Stevenage, UK) at a concentration of 10^6^ cells cm^−2^. One day after seeding, cells were incubated in the presence or absence of **PTH1**, **PTH2**and **PTH3** (10 µM) for 24 h. The incubation of cells with Hoechst 33,342 (5 μg mL^−1^, Invitrogen) was performed for nuclear staining for 15 min and cells were washed 3 times with culture medium. Fluorescence pictures were recorded on confocal microscope (LSM 780 live, Carl Zeiss Microscope) under a 450 nm wavelength excitation for polythiophenes detection and a 760 nm wavelength for nuclei imaging. A high magnification (63×/1.4 OIL DIC Plan-Apo) was used for all images.

### 2.9. Gel Electrophoresis with siRNA

A total of 5 μL of a 20 μM siRNA, mixed with the appropriate amounts of polythiophenes (in order to reach the desired P^+^/P^-^ ratio) in RNase free water (final volume: 20 μL) was used to perform gel retardation assays with siRNA and 4 μL of Blue 6X loading dye (Fisher Scientific, Hampton, NH, USA) was then added. These samples were submitted to an electrophoresis performed with a 2% wt/vol agarose gel in TBE (90 mM Tris-borate/2 mM EDTA, pH 8.2) at 50 V for 1 h. The standard was a 100 bp DNA ladder (Sigma-Aldrich, Saint-Quentin-Fallavier, France). To visualize siRNA, a GelRed nucleic acid gel stain (Interchim, Montluçon, France) was used allowing to detect siRNA through an ultraviolet transilluminator (Infinity Gel documentation Imaging, Vilber Lourmat, Paris, France).

### 2.10. In Vitro Combined PDT and siRNA Delivery

MDA-MB-231 Luc RFP cells derived from MDA-MB-231 human breast cancer cells by the stable transfection of firefly luciferase gene and RFP for red coloration and easy detection were used. They were seeded in a multiwell plate (96 well white opaque tissue culture plates) at 2 × 10^3^ cells cm^−2^ in 100 µL culture medium. Eight hours after seeding, the cells were incubated with or without 5 µM PTH2/siRNA and **PTH3**/siRNA polyplexes at a P^+^/P^−2^ ratio of 100 during the allocated 24 h. The quantities of siRNA used for PHT2 and **PTH3** complexation were 132 and 81 µM, respectively. Cells were irradiated for 5 min using a standard fluorescent microscope (Leica DM IRB) with a mercury lamp at 450 nm, magnification x4. Next day, cells were irradiated for a second time with the same process. The day after, to quantify either the cell death or the transfection efficieny of a 21-mer siRNA targeting the expression of luciferase inside MDA-MB 231 Luc RFP cells, cells were submitted to an MTT assay and to luciferase activity assay, respectively. The siRNA targeting sequence (sense: AACUUACGCUGAGUACUUCGA and anti-sense UCGAAGUACUCAGCGUAAGUU) for luciferase was purchased from Eurogentec (Serring, Belgium). The addition into a culture medium of luciferin (10^−3^ M, Promega, Charbonnières-les-bains, France) was used to determine the expression of luciferase. A total of 10 min after, a plate reader CLARIOstar^®^ High-Performance Monochromator Multimode Microplate Reader (BMG Labtech, Ortenberg, Germany) was used to measure living cell luminescence. The cytotoxicity or phototoxicity of polymers were evaluated by MTT assay.

### 2.11. Statistical Studies

Statistical analyses were carried out using student *t*-test, to compare paired groups of data. A *p*-value of <0.05 was considered to be statistically significant.

## 3. Results and Discussion

### 3.1. Polymer Synthesis

**Scheme 1:**
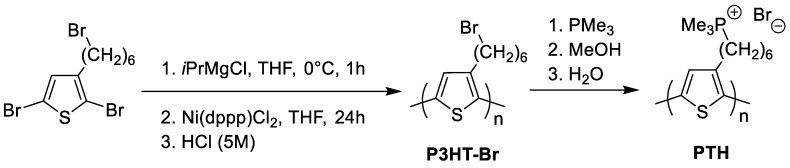
Synthetic pathway towards phosphonium-based conjugated polyelectrolytes (CPEs) (**PTH**).

The phosphonium-based polythiophene conjugated polyelectrolytes were synthesized according to our previous reported procedures [31,58]. Briefly, poly(3-(6’-bromohexyl)thiophene) precursors (**P3HT-Br**) were first synthesized by KCTCP (Scheme 1), which enables the preparation of polythiophenes with a high degree of control over the final structure and the molecular weight [50,58,60]. Indeed, due to the living chain-growth polymerization mechanism, a control on the molecular weight of the polymer can be achieved by carefully tuning the feed ratio of monomer to the nickel catalyst [50,60]. Using this method, three different well-defined polymer precursors were synthesized in view of evaluating the effect of polythiophene molecular weight on the PDT effect and gene delivery properties (Table 1). These precursors were then converted in 75–80% yield into the corresponding phosphonium-based polythiophene CPEs by reaction with trimethylphosphine (Scheme 1) [31]. The polyelectrolyte nature of these CPEs prevents direct molecular weight determination using size exclusion chromatography (SEC). Consequently, the molecular weights of these CPEs were estimated from the number of units calculated from the **P3HT-Br** precursors and the molar mass of the phosphonium repeating unit (321 g·mol^−1^) leading to M_n_ = 53 kDa (**PTH1**), M_n_ = 19 kDa (**PTH2**) and M_n_ = 11.5 kDa (**PTH3**).

### 3.2. Optical Properties of the Phosphonium-Based Polythiophene CPEs

**Figure 1:**
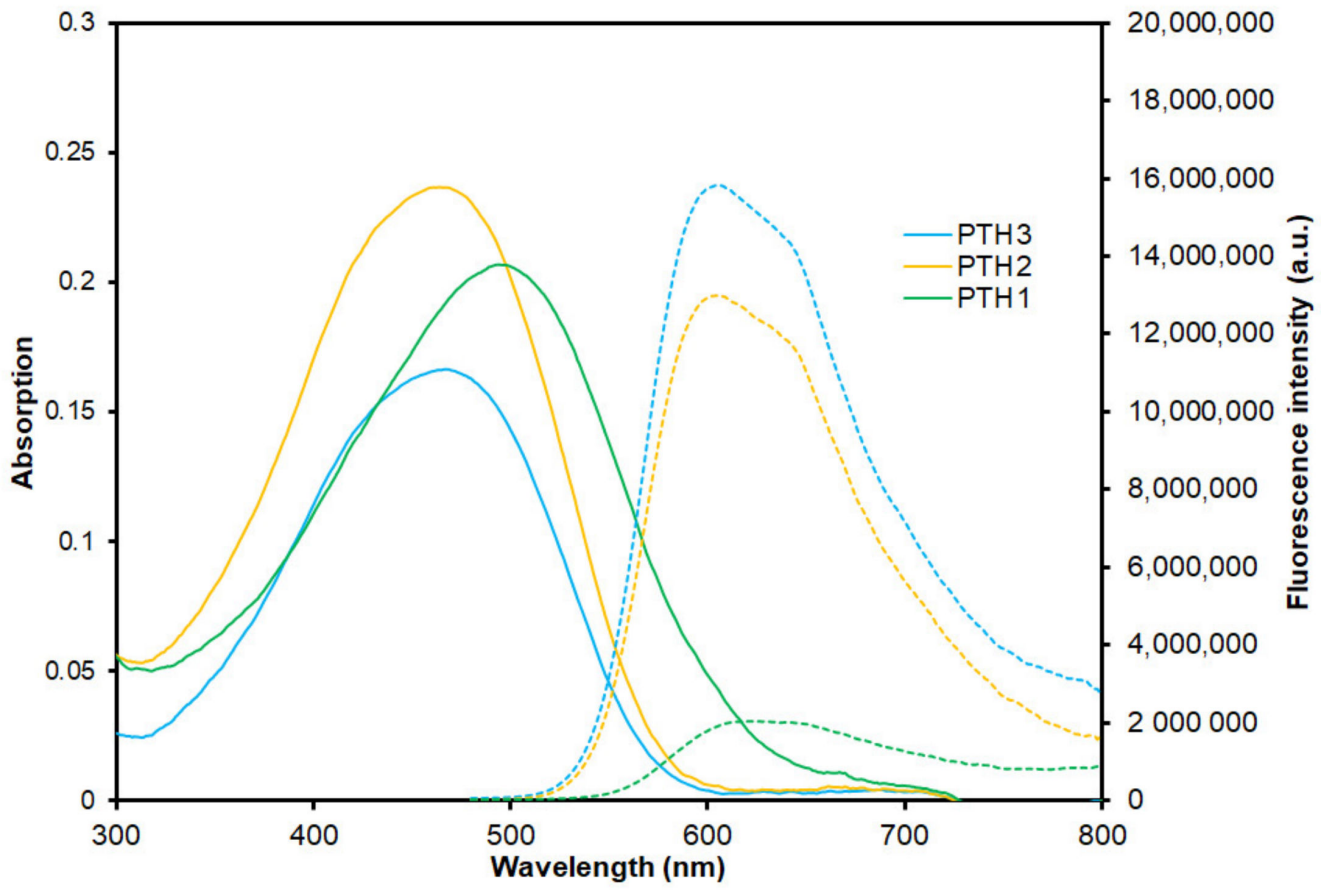
UV-vis. absorption spectra (solid line) and emission spectra (λ_exc_ = 466 nm) (dotted line) of **PTH1** (0.37 μM), **PTH2** (0.68 μM) and **PTH3** (0.81 μM) in water.

The UV-visible absorption and emission spectra of CPEs are depicted in Figure 1 and the corresponding data are summarized in Table 2.

The UV-visible absorption spectra of these CPEs show a broad absorption band attributed to a π-π* transition [63]. The maxima absorption wavelengths (λ_abs max_) are observed at ~465 nm for **PTH2** and **PTH3**, while a red-shifted absorption is noticed (495 nm) for **PTH1**, indicating a significant extent of the π-conjugation. The emission spectra of these CPEs are also dependent on the molecular weight since a maxima emission wavelength of 605 nm is found for **PTH2** and **PTH3**, whereas, for **PTH1**, an emission broad band centered at ~620 nm is observed. These results suggest that the cationic polythiophene **PTH1** adopts a more ordered and planar conformation, which may be due to the polymer aggregation in water. Indeed, compared to other CPEs, **PTH1** exhibits a lower solubility in water due to its much higher molecular weight. To gain insight into the aggregation behaviour of these polymers, the fluorescence quantum yields of the three cationic polythiophenes were measured using tris(bipyridine)ruthenium(II) chloride ([Ru(bpy)_3_]Cl_2_) in water as a reference (Φ_F_ = 0.042) [61]. As observed in Table 2, **PTH2** and **PTH3** exhibit similar fluorescence quantum yields (7–8%) [48], whereas a strong decrease of the fluorescence quantum yields is noted for **PTH1** (1%), which is consistent with a significant aggregation in solution as a result of longer π-conjugated backbone leading to increased interchain interactions and fluorescence self-quenching [64,65,66]. The increased interchain contacts in solution for **PTH1** in comparison with **PTH2** and **PTH3** are also evidenced by the decrease in the singlet state emissive lifetime (0.5 ns vs. 0.7 ns) (Table 2 and Figure S1 in the Supporting Information). These values of singlet state emissive lifetime are in good agreement with those reported for poly(3-hexylthiophene) (P3HT) [67,68]. To evaluate the degree of aggregation of these polymers, their size was then determined by Dynamic Light Scattering (DLS) leading to average diameters of around 110 and 103 nm for **PTH2** and **PTH3**, respectively, while, for **PTH1**, an average diameter of around 527 nm is observed, confirming its aggregation in aqueous media (Figures S3–S5 in the Supporting Information).

### 3.3. Cytotoxicity, Imaging and Photodynamic Therapy Activity

**Figure 2:**
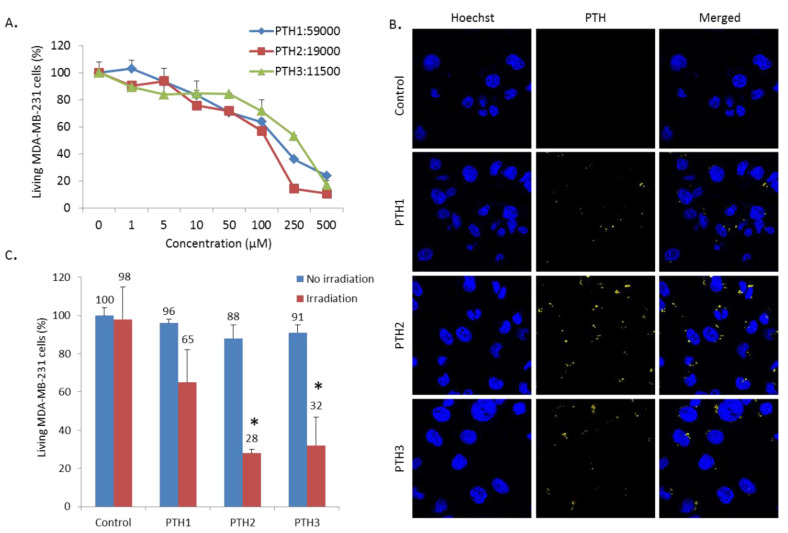
(**A**) MDA-MB-231 cells were incubated with different concentrations of **PTH1**, **PTH2** and **PTH3** during 72 h. After treatment, cells were submitted to MTT assay to quantify the cell death. (**B**) MDA-MB-231 cells were incubated with or without **PTH1**, **PTH2** and **PTH3** during the allocated 24 h. The cells were incubated with Hoechst 15 min. They were imaged at 450 nm for polythiophenes and 760 nm for Hoechst using a confocal miscroscope. (**C**) MDA-MB-231 cells were incubated with **PTH1**, **PTH2** and **PTH3** during the allocated 24 h. The cells were irradiated for 5 min using a standard fluorescent microscope with a mercury lamp at 450 nm, magnification x4. Two days after irradiation, cells were submitted to MTT assay to quantify the cell death. * Statistical significance (*p* < 0.05) of the irradiated condition versus the non-irradiated condition using Student’s *t*-test.

To demonstrate the in vitro biocompatibility of phosphonium-based polythiophene CPEs, their cytotoxicity against MDA-MB-231 cells were estimated using the 3-[4,5-dimethylthiazol-2-yl]-2,5-diphenyl tetrazolium bromide (MTT) method, in which soluble MTT is converted into formazan crystal by mitochondrial dehydrogenases, thus allowing one to measure the cell viability. MDA-MB-231 cells were grown in 96-well culture plates and were then treated with **PTH1**, **PTH2** and **PTH3** for 72 h. Figure 2A shows the effect of **PTH1**, **PTH2** and **PTH3** with concentrations ranging from 0 to 500 μM (relative to repeating unit) on the viability of MDA-MB-231 cells. Although these cationic polymers displayed in vitro toxicity from a concentration of 10 μM, the cell viability is still larger than 70% at a concentration of 50 μM. These values are in good agreement with those previously reported for other CPEs, such as cationic polyfluorenes [38] and poly(fluorene-co-thiophenes) [69]. From Figure 2A, the half inhibitory concentrations (IC50) values for these cationic polymers were also determined leading respectively to IC50 > 100 μM for **PTH1** and **PTH2** and IC50 > 250 μM for **PTH3**. Based on these results, cationic polymers were then used at a concentration of 5 μM for investigating PDT and gene delivery applications.

Considering PDT applications, we first evaluated the ability of phosphonium-based polythiophene CPEs to generate singlet oxygen (^1^O_2_) by monitoring photoluminescence of ^1^O_2_ at 1270 nm in aerated D_2_O using Ru(bpy)_3_ as a reference (Φ_Δ_ = 0.53) (Figure S2 in the Supporting Information) [62]. **PTH2** and **PTH3** showed relatively low ^1^O_2_ luminescence quantum yield: Φ_Δ_ = 0.12 and = 0.14, respectively, while **PTH1** did not produce ^1^O_2_ likely due to aggregation in solution resulting in increased interchain contacts and thus intermolecular charge transfer. Indeed, aggregation is known to lead to reduced ROS generation as previously observed for conventional PSs, such as porphyrins in aqueous media [70,71]. Then, we explored their cellular uptake by MDA-MB-231 cells. The internalization of **PTH1**, **PTH2** and **PTH3** in MDA-MB-231 cells was clearly visualized by fluorescence microscopy (Figure 2B) exploiting the fluorescence properties of phosphonium-based polythiophene CPEs. Indeed, the cationic charge of the phosphonium side groups of these polythiophene-based CPEs enables their binding to the negatively charged cell membrane and subsequently enhances their endocytosis by cells. As shown in Figure 2B, the orange fluorescence of **PTH1**, **PTH2** and **PTH3** indicated that they are mainly accumulated around the perinuclear region (located concurrently by Hoechst staining) [72]. Finally, the efficiency of CPEs as PSs towards MDA-MB-231 cells was evaluated in vitro under illumination.

To this aim, MDA-MB-231 cells were incubated for 24 h with phosphonium-based polythiophene CPEs at 5 μM and then, submitted or not to monophotonic light excitation at 450 nm for 5 min. An MTT assay was performed two days after illumination to determine the cells viability. As indicated in Figure 2C, none of the cationic polymers showed significant cytoxicity in the dark (between 4 and 12% cell death at 5 μM). After illumination at 450 nm for 5 min, **PTH2** and **PTH3** exhibits efficient phototoxicity, inducing around 60% cell death, whereas, in these conditions, no significant phototoxicity is noted for **PTH1** due to its aggregation in aqueous media and thus its incapacity to generate ^1^O_2_ under illumination.

### 3.4. In Vitro Combined PDT and siRNA Delivery

**Figure 3:**
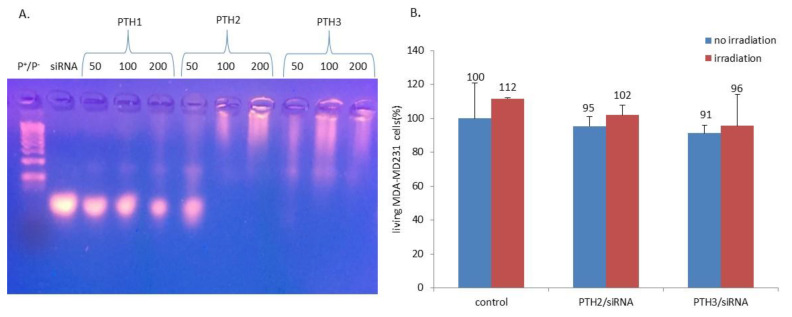
(**A**) Gel electrophoresis analysis showing the complexation of siRNA by the polythiophenes under study. (**B**) MDA-MB-231 were incubated with **PTH2**/siRNA and **PTH3**/siRNA during the allocated 24 h. The cells were illuminated during 5 min using standard fluorescent microscope with a mercury lamp at 450 nm, magnification x4. Two days after illumination, cells were submitted to an MTT assay to quantify living cells.

The siRNA loading capability of **PTH1**, **PTH2** and **PTH3** was first studied. For this, an agarose-gel electrophoresis assay was performed to estimate the siRNA complexation efficiency of these cationic polythiophene according to the P^+^/P^-^ molar ratio between the positively charged phosphonium groups (P^+^) in the polymer and the negatively charged phosphodiester groups (P^−^). As shown in Figure 3A, as the P^+^/P^-^ ratio increases, the negative charges of siRNA were gradually neutralized and moved to a positive electrode (retardation effect), except for **PTH1**, in which, regardless of the used P^+^/P^-^ ratio, the siRNA migration was not inhibited. In the case of **PTH2** and **PTH3**, the migration of siRNA was inhibited at P^+^/P^-^ ratio of 100 and 50, respectively. Consequently, we decided to focus only on cationic polythiophenes **PTH2** and **PTH3** for the further combination of in vitro PDT and gene delivery since **PTH1** did not generate ^1^O_2_ and was not able to complex siRNA. A P^+^/P^-^ ratio of 100 was chosen for **PTH2** and **PTH3** for the further studies of the in vitro combined PDT and siRNA delivery. The size of the **PTH2/siRNA** and **PTH3/siRNA** polyplexes at a P^+^/P^-^ ratio of 100 was then determined by Dynamic Light Scattering leading to polyplexes with average diameters of around 82 and 92 nm, respectively (Figures S6 and S7 in the Supporting Information), a suitable size for facilitating endocytosis [73,74]. Furthermore, the positive zeta potential of **PTH2**/siRNA (ζ = 15 mV) and **PTH3**/siRNA (ζ = 20 mV) polyplexes at a P^+^/P^-^ ratio of 100 allows these polyplexes to interact with the negatively charged cell membrane favorizing their cellular uptake (Figure S5 and S6 in the Supporting Information) [40,75]. The in vitro PDT activity of **PTH2/**siRNA and **PTH3**/siRNA nanoparticles was first examined, exploiting the same conditions as described above. As shown in Figure 3B, siRNA complexation by cationic polythiophenes **PTH2** and **PTH3** led to the complete loss of the PDT activity of the polymers since no phototoxicity was noticed upon illumination, while, for polymers alone, around 60% cell death was observed. We speculate that this loss of PDT activity can be attributed to the formation of aggregates and thus fluorescence self-quenching and reduced ROS production [32,44].

**Figure 4:**
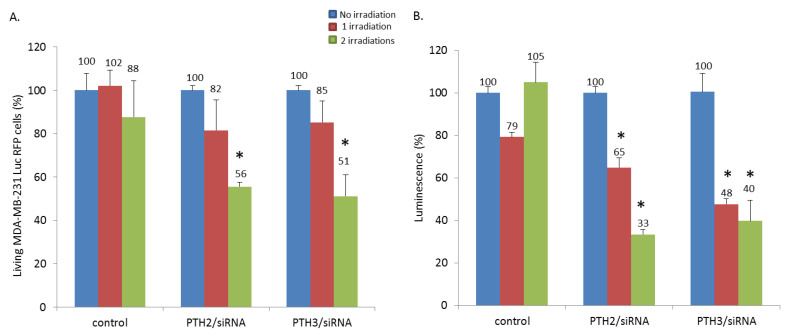
(**A**) MDA-MB-231 Luc RFP cells were incubated with **PTH2**/siRNA and **PTH3**/siRNA at a P^+^/P^-^ ratio of 100 during 24 h. The cells were irradiated for 5 min using a standard fluorescent microscope with a mercury lamp at 450 nm, magnification x4. After 24 h, the cells were irradiated a second time with the same process. The day after, the cells were submitted to an MTT assay to quantify the cell death. (**B**) Luciferase activity assay showing the transfection of a 21-mer siRNA targeting the expression of luciferase inside MDA-MB-231 Luc RFP cells. The experiments were carried out with **PTH2**/siRNA and **PTH3**/siRNA at a P^+^/P^-^ ratio of 100 without or after one or two irradiations (450 nm, 5 min). * Statistical significance (*p* < 0.05), of the irradiated condition versus non irradiated condition using Student’s *t*-test.

Finally, to evaluate the therapeutic potential of these polymers in PDT-siRNA combination therapy, we set up experiments using siRNA targeting the expression of luciferase gene in MDA-MB-231 Luc RFP cells. MDA-MB-231 Luc RFP cells were incubated for 24 h with **PTH2**/siRNA and **PTH3**/siRNA polyplexes at a P^+^/P^-^ ratio of 100 at 5 μM and then submitted or not to one or two monophotonic light irradiations at 450 nm for 5 min, with the second light irradiations occuring 24 h after the first one. Figure 4 shows the obtained results for in vitro combined PDT and siRNA delivery exploiting **PTH2**/siRNA and **PTH3**/siRNA polyplexes in MDA-MB-231 Luc RFP cells. As observed previously in Figure 3B, the first light irradiation did not result in cell death, as confirmed in Figure 4A (red bars). However, it enables the delivery of siRNA targeting the luciferase gene inside **MDA-MB 231 Luc RFP** cells leading to 35% and 52% of luciferase gene silencing for **PTH2** and **PTH3**, respectively (Figure 4B). The siRNA decomplexation is confirmed by the restoration of the PDT activity of **PTH2**/siRNA and **PTH3**/siRNA leading, respectively, to 44% and 49% of cell death after a second light irradiation (Figure 4A). This second light irradiation (green bars) also results in a significant improvement in the luciferase gene silencing, mainly for **PTH2**/siRNA leading to a 67% decrease in luminescence (Figure 4B). We can note that, for **PTH3**/siRNA, the effect on luminescence decrease is maximal after only one irradiation (48% at the first irradiation and 40% after 2 irradiations), suggesting that **PTH3** can be more easily used for nucleic acid delivery. This light-triggered siRNA release will necesitate deeper investigation, and the understanding of the mechanism responsible for this effect will help us to develop highly potent polythiophenes for nucleic acid delivery under light stimulation. All in all, these results show the remarkable synergistic effect of using these cationic polymers both as a polymer vector for siRNA delivery and as a PS for PDT.

## 4. Conclusions

Phosphonium-based conjugated polythiophenes with different molecular weight (going from 11.5 kDa to 53 kDa) were prepared by KCTCP and used as multifunctional platforms to complex and deliver siRNA and to generate ROS species for PDT. Upon light irradiation, cationic polythiophenes **PTH2**and **PTH3** with lower molecular weight were able to light-sensitize surrounding oxygen into ROS, which could disturb the endosome-lysosome membrane and thus lead to an enhanced endosomal escape and more efficient gene delivery. In contrast, these polymers are also fluorescent, allowing for the imaging of intracellular location through confocal microscopy; thus, their good internalization was confirmed. The most promising conjugated polyelectrolytes, **PTH2**and **PTH3**, were then used as carriers for siRNA delivery. Due to their cationic and amphipathic features, these polymers were found to effectively self-assemble with siRNA, deliver siRNA targeting the luciferase gene in MDA-MB-231 cancer cells expressing luciferase and lead to 35 and 52% of the gene silencing effect. In parallel, the photodynamic activity of these cationic polymers was restored after siRNA delivery, demonstrating their potential for combined PDT and gene therapy. Nevertheless, further investigation will be needed to determine the mechanism responsible for the light-triggered release of siRNA by these cationic polythiophenes. This study illustrates that multiple functions can be achieved by exploiting the features of conjugated polyelectrolytes and suitably tailoring their molecular structures (molecular weight, nature of the ionic group, etc.). To go further, modifying the polymer structure by introducing notably multiple cationic side groups per monomeric units will be necessary to improve their ability to bind and condense nucleic acid and their solubility in aqueous medium, which will thus positively impact their optical properties. In addition, two-photon excited photodynamic therapy and siRNA delivery experiments will be conducted in the future to extend the scope of this multifunctional platform.

## Figures and Tables

**Table 1:** Characteristics of the synthesized **P3HT-Br** precursors.

Name	Yield (%)	M_n_ (g·mol^−1^) ^1^	Ð	DP ^2^
**P3HT-Br1**	55	40 600	1.16	165
**P3HT-Br2**	72	14 500	1.28	59
**P3HT-Br3**	62	8 500	1.31	36

^1^ M_n_ determined by SEC in THF except for **P3HT-Br3**, which was performed in CHCl_3_. ^2^ Degree of polymerization calculated from M_n_.

**Table 2:** Optical characteristics of phosphonium-based polythiophene CPEs in water.

Compound	λ_abs max_ (nm)	λ_em max_ (nm) ^1^	Φ_F_^2^	τ_F_ (ns)	ΦΔ
**PTH1**	495	624	0.01	0.5	< 0.01
**PTH2**	464	605	0.07	0.7	0.13
**PTH3**	466	605	0.08	0.7	0.15

^1^ λ_exc_ = 466 nm, ^2^ measured using tris(bipyridine)ruthenium(II) chloride [Ru(bpy)_3_]Cl_2_ in water as a reference (Φ_F_ = 0.042) [61], ^3^ measured using [Ru(bpy)_3_]Cl_2_ in D_2_O as a reference (ΦΔ = 0.53) [62].

## Supplementary Materials

The following are available online at https://www.mdpi.com/2079-4991/10/8/1432/s1, Figure S1: Fluorescence decay of **PTH1** (0.37 μM), **PTH2** (0.68 μM) and **PTH3** (0.81 μM) in water in D_2_O (λ_exc_ = 407 nm), Figure S2: Singlet Oxygen emission spectra of **PTH1**, **PTH2** and **PTH3** in D_2_O (λ_exc_ = 466 nm), Figure S3: Particle Size distribution of **PTH1** in water (5 μM) at 25 °C, Figure S4: Particle Size distribution of **PTH2** in water (5 μM) at 25 °C, Figure S5: Particle Size distribution of **PTH3** in water (5 μM) at 25 °C, Figure S6: Particle size distribution (up) and zeta potential (down) of **PTH2**/siRNA polyplex in water (5 μM) at a P^+^/P^-^ ratio of 100 at 25 °C, Figure S7: Particle size distribution (up) and zeta potential (down) of **PTH3**/siRNA polyplex in water (5 μM) at a P^+^/P^-^ ratio of 100 at 25 °C. Figure S8: (**A**) MDA-MB-231 cells were incubated with **PTH1**, **PTH2**, **PTH3** during 72 h. Cells were irradiated using confocal microscope with a chameleon laser beam at 800 nm, magnification x10, during 3 x 1.57 seconds. Two days after irradiation, cells were submitted to a MTT assay to quantify the cell death. (**B**) MDA-MB-231 cells were incubated with or without **PTH1**, **PTH2**, **PTH3** during 24 h. Cells were incubated with Hoechst 15 min. They were imaged at 800 nm for polythiophenes and 760 nm for Hoechst using confocal microscope.
