# Supplementary material for: Polythiophenes with Cationic Phosphonium Groups as Vectors for Imaging, siRNA Delivery, and Photodynamic Therapy
Source: Nanomaterials (Basel). 2020 Jul 22;10(8):1432. doi: 10.3390/nano10081432 (PMC7466636; doi:10.3390/nano10081432)
Supplement: Supplementary file 1 [file nanomaterials-10-01432-s001.pdf]

Supporting Information

Polythiophenes with Cationic Phosphonium Groups as Vectors for Imaging, siRNA Delivery, and Photodynamic Therapy

Laure Lichon [1], Clément Kotras [2,3], Bauyrzhan Myrzakhmetov [4], Philippe Arnoux [4], Morgane Daurat [5], Christophe Nguyen [1], Denis Durand [1], Karim Bouchmella [3], Lamiaa Mohamed Ahmed Ali [1,6], Jean-Olivier Durand [3], Sébastien Richeter [3], Céline Frochot [4], Magali Gary-Bobo [1,*], Mathieu Surin [2] and Sébastien Clément [3,*]

[1] IBMM, University of Montpellier, CNRS, ENSCM, 34093 Montpellier, France; laure.lichon@umontpellier.fr (L.L.); christophe.nguyen@umontpellier.fr (C.N.); denis.durand@umontpellier.fr (D.D.); lamiaa.ali@umontpellier.fr (L.M.A.A.)

[2] Center of Innovation and Research in Materials and Polymers (CIRMAP), University of Mons—UMONS, 20 Place du Parc, 7000 Mons, Belgium; clement.kotras@umons.ac.be (C.K.); mathieu.surin@umons.ac.be (M.S.)

[3] ICGM, University of Montpellier, CNRS, ENSCM, CC1701, Place Eugène Bataillon, 34095 Montpellier, France; karim.bouchmella@umontpellier.fr (K.B.); jean-olivier.durand@umontpellier.fr (J.-O.D.); sebastien.richeter@umontpellier.fr (S.R.)

[4] Laboratoire Réactions et Génie des Procédés (LRGP), UMR 7274, Université de Lorraine, CNRS, 54000 Nancy, France; Bauyrzhan.myrzakhmetov@univ-lorraine.fr (B.M.); Philippe.Arnoux@univ-lorraine.fr (P.A.); celine.frochot@univ-lorraine.fr (C.F.)

[5] NanoMedSyn, 15 Avenue Charles Flahault, 34093 Montpellier, France; morgane.daurat@umontpellier.fr

[6] Department of Biochemistry, Medical Research Institute, University of Alexandria, Alexandria 21561, Egypt

* Correspondence: magali.gary-bobo@inserm.fr (M.G.-B.); sebastien.clement1@umontpellier.fr (S.C.); Tel.: +33(0)-4-11-75-96-17 (M.G.-B.); +33(0)-4-67-16-14-39-71 (S.C.)

Table of contents

Figure S1: Fluorescence decay of **PTH1** (0.37 µM), **PTH2** (0.68 µM) and **PTH3** (0.81 µM) in water in D_2O (λ_{exc} = 407 nm) S2

Figure S2. Singlet oxygen emission spectra of **PTH1**, **PTH2** and **PTH3** in D_2O (λ_{exc} = 466 nm) S2

Figure S3. Particle Size distribution of **PTH1** in water (5 µM) at 25°C. S3

Figure S4. Particle Size distribution of **PTH2** in water (5 µM) at 25°C. S3

Figure S5. Particle Size distribution of **PTH3** in water (5 µM) at 25°C. S4

Figure S6. Particle size distribution (up) and zeta potential (down) of **PTH2/siRNA** polyplex in water (5 µM) at a P^+/P^- ratio of 100 at 25°C. S5

Figure S7. Particle size distribution (up) and zeta potential (down) of **PTH3/siRNA** polyplex in water (5 µM) at a P^+/P^- ratio of 100 at 25°C. S6

Figure S8. (**A**) **MDA-MB-231** cells were incubated with **PTH1**, **PTH2**, **PTH3** during 72 h. Cells were irradiated using confocal microscope with a chameleon laser beam at 800 nm, magnification x10, during 3 x 1.57 seconds. Two days after irradiation, cells were submitted to a MTT assay to quantify the cell death. (**B**) **MDA-MB-231** cells were incubated with or without **PTH1**, **PTH2**, **PTH3** during 24 h. Cells were incubated with Hoechst 15 min. They were imaged at 800 nm for polythiophenes and 760 nm for Hoechst using confocal microscope. S7

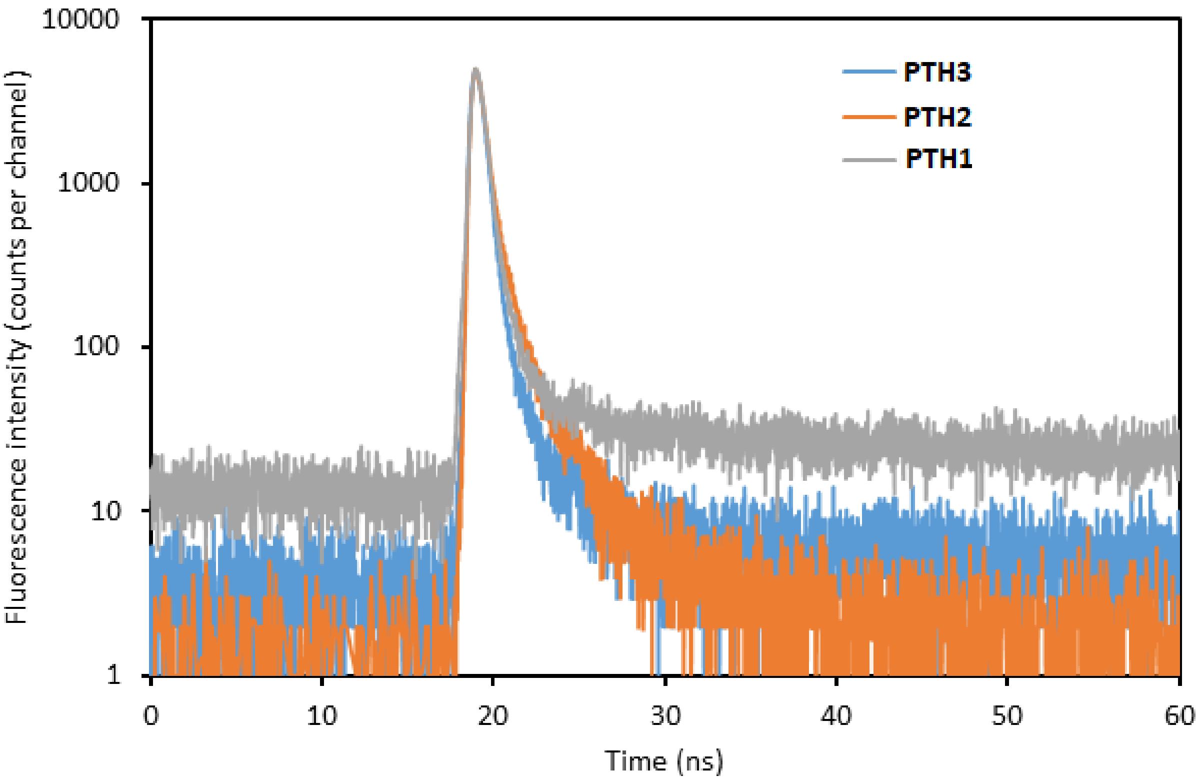

Figure S1. Fluorescence decay of **PTH1** (0.37 µM), **PTH2** (0.68 µM) and **PTH3** (0.81 µM) in water in D_2O (λ_{exc} = 407 nm).

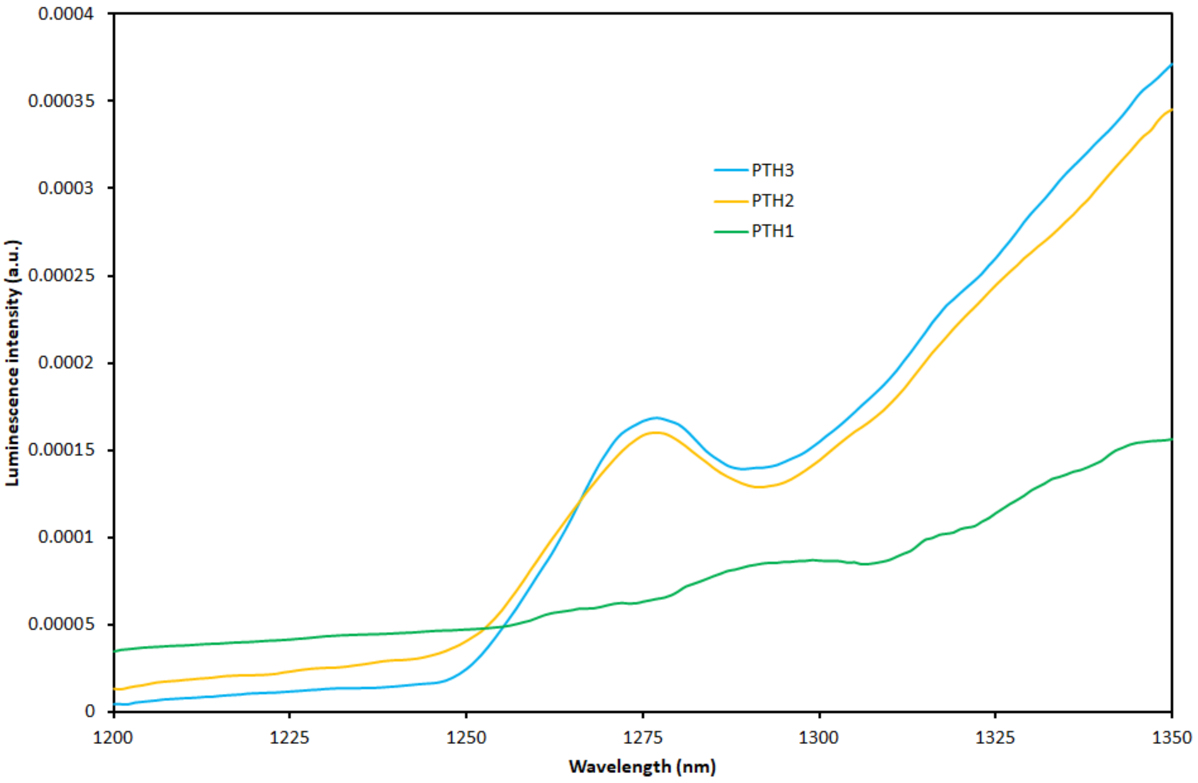

Figure S2. Singlet oxygen emission spectra of **PTH1**, **PTH2** and **PTH3** in D_2O (λ_{exc} = 466 nm).

			Size (d.nm...	% Intensity:	St Dev (d.n...
**Z-Average (d.nm):**	1647	**Peak 1:**	526,6	100,0	51,88
**PdI:**	0,536	**Peak 2:**	0,000	0,0	0,000
**Intercept:**	0,882	**Peak 3:**	0,000	0,0	0,000

Result quality **Refer to quality report**

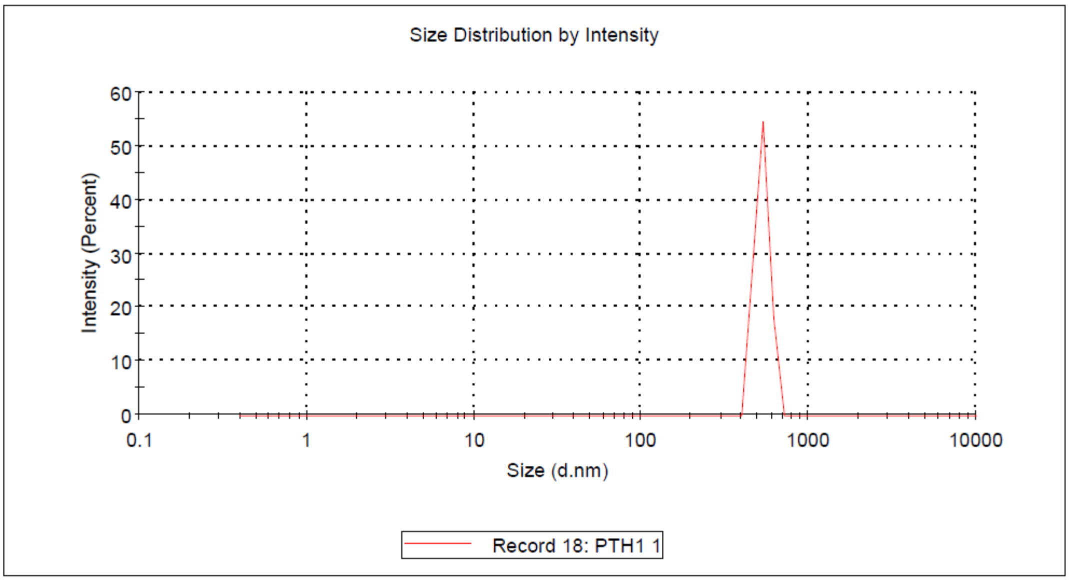

Figure S3. Particle Size distribution of **PTH1** in water (5 µM) at 25°C.

			Size (d.nm...	% Intensity:	St Dev (d.n...
**Z-Average (d.nm):**	110,3	**Peak 1:**	129,6	81,1	80,85
**PdI:**	0,386	**Peak 2:**	847,6	18,9	418,2
**Intercept:**	0,760	**Peak 3:**	0,000	0,0	0,000

Result quality **Good**

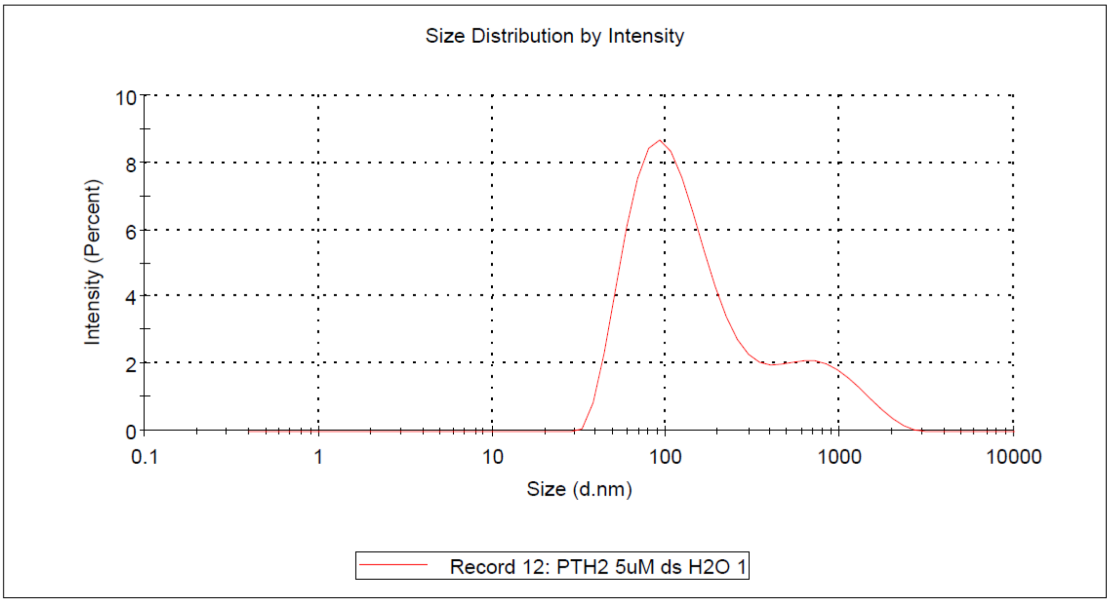

Figure S4. Particle Size distribution of **PTH2** in water (5 µM) at 25°C.

			Size (d.n...	% Number:	St Dev (d.n...
**Z-Average (d.nm):**	102,6	**Peak 1:**	48,67	100,0	16,81
**PdI:**	0,327	**Peak 2:**	0,000	0,0	0,000
**Intercept:**	0,648	**Peak 3:**	0,000	0,0	0,000
**Result quality**	**Good**				

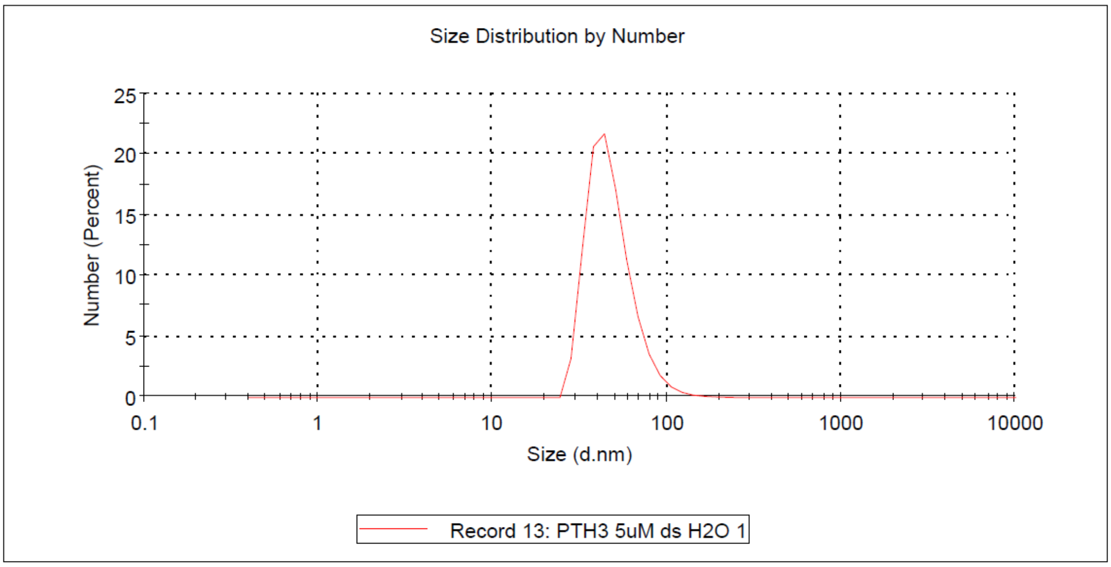

Figure S5. Particle Size distribution of **PTH3** in water (5 µM) at 25°C.

**Z-Average (d.nm):**	82,12	**Peak 1:**	115,5	100,0	70,74
**PdI:**	0,248	**Peak 2:**	0,000	0,0	0,000
**Intercept:**	0,900	**Peak 3:**	0,000	0,0	0,000
**Result quality**	**Good**				

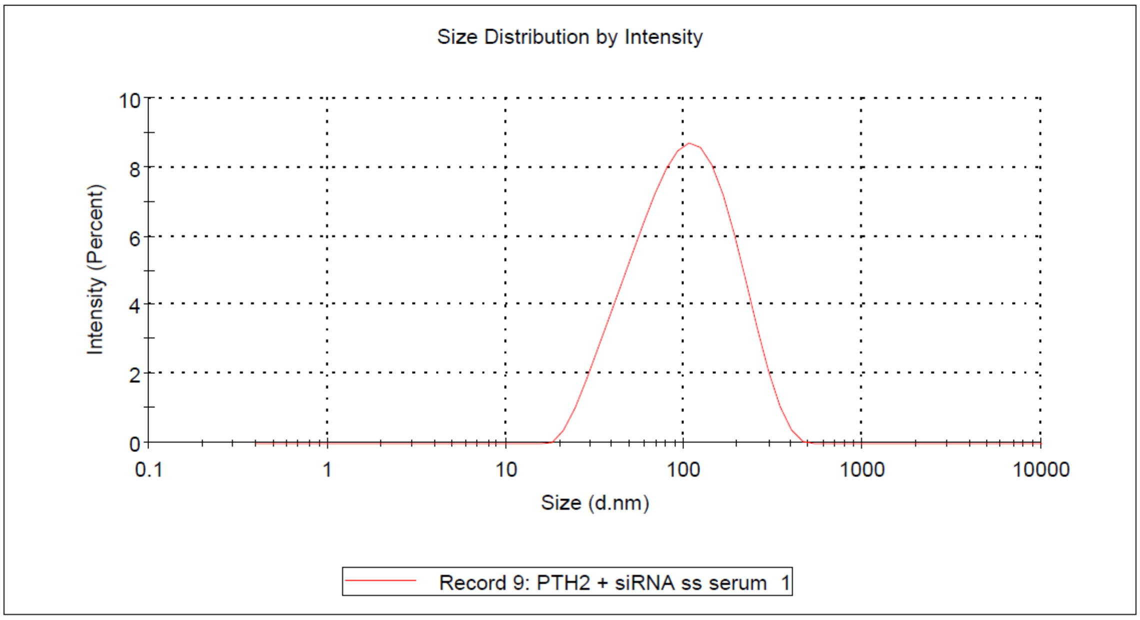

			Mean (mV)	Area (%)	St Dev (mV)
**Zeta Potential (mV):**	15,2	**Peak 1:**	15,6	98,4	10,4
**Zeta Deviation (mV):**	11,1	**Peak 2:**	-20,0	1,6	3,68
**Conductivity (mS/cm):**	0,0508	**Peak 3:**	0,00	0,0	0,00

Result quality See result quality report

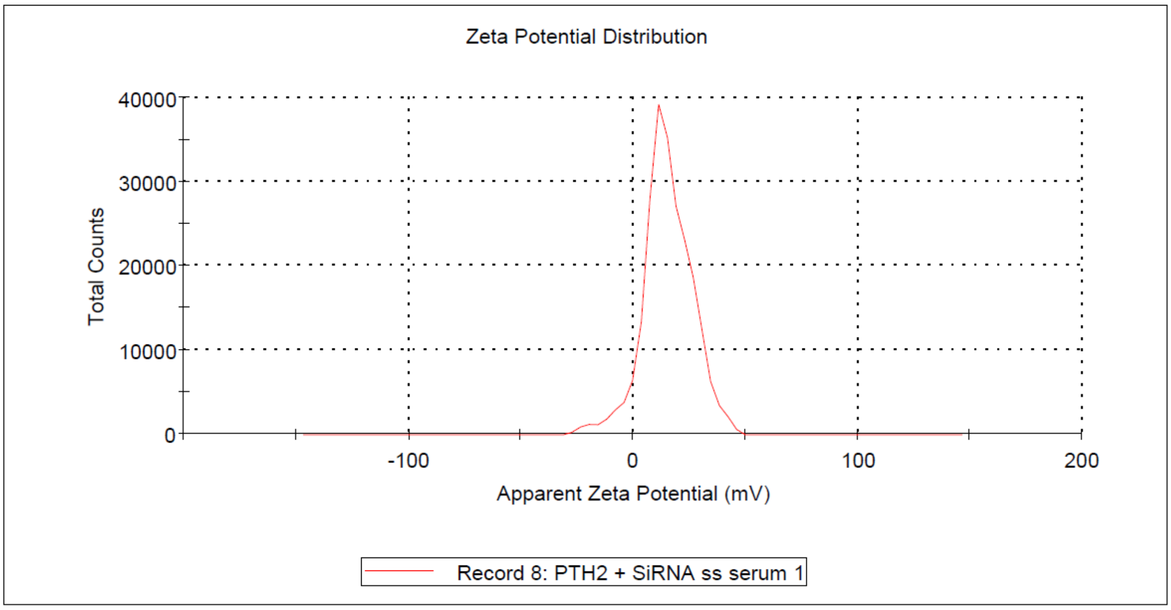

Figure S6. Particle size distribution (up) and zeta potential (down) of **PTH2/siRNA** polyplex in water (5 µM) at a P^+/P^- ratio of 100 at 25°C.

			Size (d.n...	% Number:	St Dev (d.n...
**Z-Average (d.nm):**	92,43	**Peak 1:**	22,43	100,0	7,803
**PdI:**	0,433	**Peak 2:**	0,000	0,0	0,000
**Intercept:**	0,722	**Peak 3:**	0,000	0,0	0,000

Result quality Good

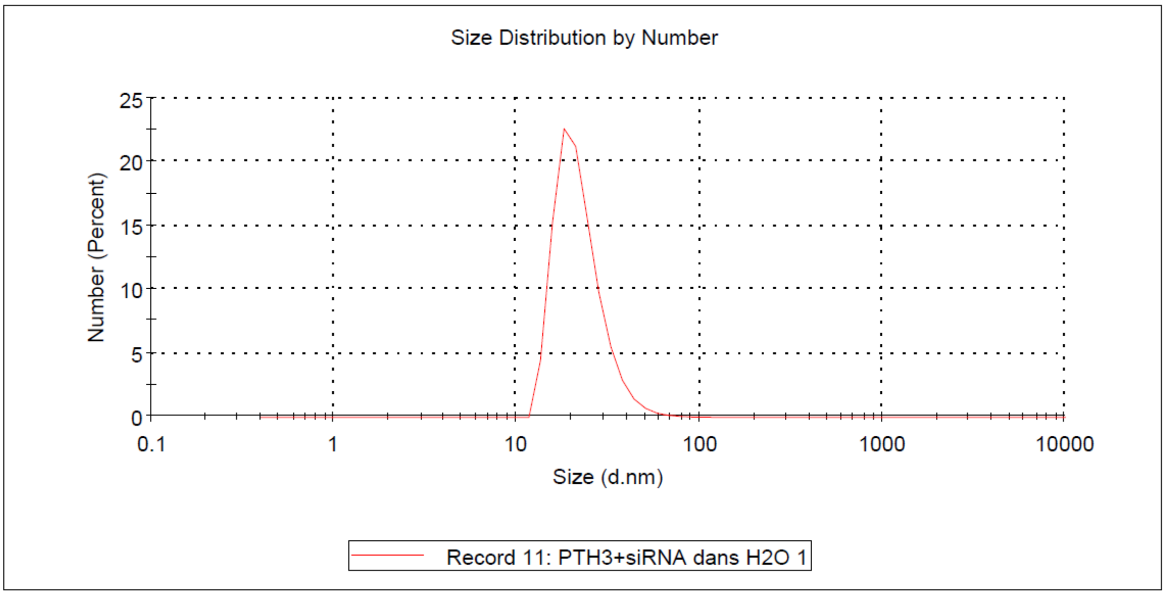

			Mean (mV)	Area (%)	St Dev (mV)
**Zeta Potential (mV):**	20,2	**Peak 1:**	19,6	96,0	10,4
**Zeta Deviation (mV):**	12,0	**Peak 2:**	51,0	3,2	5,44
**Conductivity (mS/cm):**	0,0739	**Peak 3:**	-26,9	0,6	2,05
**Result quality**	**See result quality report**				

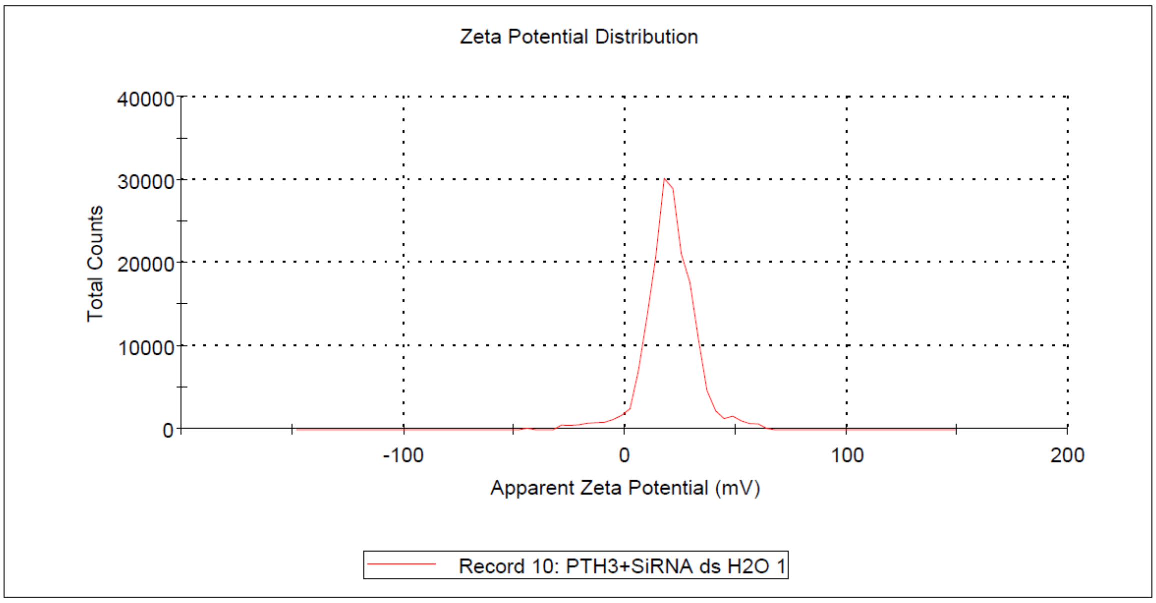

Figure S7. Particle size distribution (up) and zeta potential (down) of **PTH3/siRNA** polyplex in water (5 µM) at a P^+/P^- ratio of 100 at 25°C.

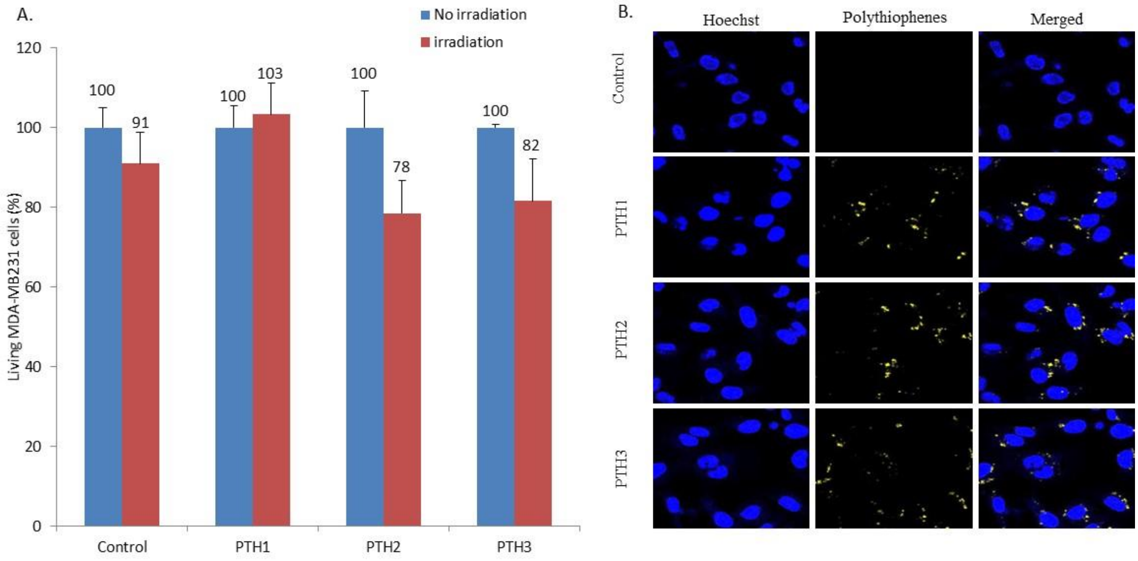

Figure S8. (**A**) **MDA-MB-231** cells were incubated with **PTH1**, **PTH2**, **PTH3** during 72 h. Cells were irradiated using confocal microscope with a chameleon laser beam at 800 nm, magnification x10, during 3 x 1.57 seconds. Two days after irradiation, cells were submitted to a MTT assay to quantify the cell death. (**B**) **MDA-MB-231** cells were incubated with or without **PTH1**, **PTH2**, **PTH3** during 24 h. Cells were incubated with Hoechst 15 min. They were imaged at 800 nm for polythiophenes and 760 nm for Hoechst using confocal microscope.
